# Dynamic cerebral autoregulation is attenuated in young fit women

**DOI:** 10.14814/phy2.13984

**Published:** 2019-01-16

**Authors:** Lawrence Labrecque, Kevan Rahimaly, Sarah Imhoff, Myriam Paquette, Olivier Le Blanc, Simon Malenfant, Audrey Drapeau, Jonathan D. Smirl, Damian M. Bailey, Patrice Brassard

**Affiliations:** ^1^ Department of Kinesiology Faculty of Medicine Université Laval Québec Canada; ^2^ Research center of the Institut universitaire de cardiologie et de pneumologie de Québec Québec Canada; ^3^ Concussion Research Laboratory Health and Exercise Sciences University of British Columbia Okanagan British Columbia Canada; ^4^ Neurovascular Research Laboratory Faculty of Life Sciences and Education University of South Wales South Wales United Kingdom

**Keywords:** Brain, cerebral blood flow, cerebral pressure–flow relationship, dynamic cerebral autoregulation, sex differences

## Abstract

Young women exhibit higher prevalence of orthostatic hypotension with presyncopal symptoms compared to men. These symptoms could be influenced by an attenuated ability of the cerebrovasculature to respond to rapid blood pressure (BP) changes [dynamic cerebral autoregulation (dCA)]. The influence of sex on dCA remains unclear. dCA in 11 fit women (25 ± 2 years) and 11 age‐matched men (24 ± 1 years) was compared using a multimodal approach including a sit‐to‐stand (STS) and forced BP oscillations (repeated squat‐stand performed at 0.05 and 0.10 Hz). Prevalence of initial orthostatic hypotension (IOH; decrease in systolic ≥ 40 mmHg and/or diastolic BP ≥ 20 mmHg) during the first 15 sec of STS was determined as a functional outcome. In women, the decrease in mean middle cerebral artery blood velocity (MCAv_mean_) following the STS was greater (−20 ± 8 vs. −11 ± 7 cm sec^−1^; *P = *0.018) and the onset of the regulatory change (time lapse between the beginning of the STS and the increase in the conductance index (MCAv_mean_/mean arterial pressure) was delayed (*P = *0.007). Transfer function analysis gain during 0.05 Hz squat‐stand was ~48% higher in women (6.4 ± 1.3 vs. 3.8 ± 2.3 cm sec^−1^ mmHg^−1^; *P = *0.017). Prevalence of IOH was comparable between groups (women: 4/9 vs. men: 5/9, *P = *0.637). These results indicate the cerebrovasculature of fit women has an attenuated ability to react to rapid changes in BP in the face of preserved orthostasis, which could be related to higher resting cerebral blood flow allowing women to better face transient hypotension.

## Introduction

The prevalence of orthostatic hypotension is higher in young women compared to men (Fu et al. 2004). In addition, young women suffer more often from symptoms of cerebral hypoperfusion such as light‐headedness, nausea, and blurred vision (Ali et al. 2000). These symptoms could be influenced by an attenuated ability of the cerebrovasculature to respond to rapid changes in arterial blood pressure (BP) [traditionally referred to as dynamic cerebral autoregulation (dCA)].

Accumulating evidence supports the notion that cerebral blood flow (CBF) is regulated differently in women compared to men and dependent upon age (Aanerud et al. 2017). Resting CBF (Marinoni et al. 1998) and cerebrovascular reactivity to carbon dioxide (Kastrup et al. 1997) are higher in women. Although some disparities seem to exist in regards to CA (Wang et al. 2010), very few studies have attempted to assess this crucial CBF determinant in healthy young women. Using transfer function analysis (TFA) of spontaneous or forced oscillations in mean arterial pressure (MAP) and middle cerebral artery blood velocity (MCAv), investigators reported older women have either similar (Patel et al. 2016) or enhanced dCA (Edgell et al. 2012; Deegan et al. 2011; Xing et al. 2017) compared to age‐matched men. Of note, older women present less orthostatic symptoms or syncope than their younger counterparts (Romme et al. 2008). Nevertheless, these findings are difficult to translate to a younger population since previous findings have shown older women regulate CBF in a different manner when compared with younger women (Edgell et al. 2012).

Most metrics quantifying dCA are generally unrelated to each other (Tzeng et al. 2012). Furthermore, characterization of dCA employing diverse analytical techniques can produce variable physiological interpretations (Tzeng et al. 2012; Tzeng and Ainslie 2014). Thus, when performing investigations of dCA, the utilization of a multimetrics approach could help improve our understanding of this response. Previously, we have employed this approach to study the influence of cardiorespiratory fitness (CRF) on dCA in healthy fit men (Labrecque et al. 2017). This approach revealed CRF is associated with an intact ability of the cerebrovasculature to dampen spontaneous oscillations in MAP (comparable TFA metrics between fit men vs. controls). This approach also included forced BP oscillations using repeated squat‐stand maneuvers, to improve the interpretation of the linear association between BP and MCAv (Claassen et al. 2009), which revealed a reduced capability of reacting to large and rapid changes in MAP in the trained state (delayed onset of the regulatory response; increased absolute TFA gain during 0.10 Hz repeated squat‐stand maneuvers). These results highlight the importance of including BP stimuli of different natures and magnitudes when examining the capability of the cerebrovasculature to respond to changes in MAP (Simpson and Claassen 2018).

Therefore, the aim of this study was to examine to what extent sex potentially influences dCA in a young and fit population using a multiple assessment strategy and hemodynamic stressors (sit‐to‐stand and TFA of forced MAP and MCAv oscillations). We also determined the prevalence of initial orthostatic hypotension (IOH) as a functional outcome, in order to appreciate how the potential impact of sex on dCA translates in terms of physiological outcome. We hypothesized men would have better dCA compared to women, and dCA metrics would be related to IOH.

## Materials and Methods

### Ethics and informed consent

All participants provided informed consent prior to participating in the investigation, and the study was approved by the Comité d’éthique de la recherche de l'IUCPQ‐Université Laval (CER: 20869 and 21180).

### Participants

Twenty‐two moderately trained endurance athletes were recruited for this study: eleven women [peak oxygen consumption (V̇O_2peak_): 48.1 ± 4.1 mL·kg·min^−1^] and eleven men (V̇O_2peak_: 56.8 ± 4.4 mL·kg·min^−1^; *P < *0.001 vs. women). Women and men were matched for age, body mass index (BMI), and volume of weekly training. All the participants competed in a variety of endurance‐based sports including cycling (women: *n *= 1; men: *n *= 5), triathlon (women: *n *= 4; men: *n *= 5), mountain biking (women: *n *= 1), running (women: *n *= 4), and cross‐country skiing (women: *n *= 1; men: *n *= 1). All participants were free from any medical conditions, demonstrated a normal 12‐lead ECG, and were not taking any medications. Two women were taking oral contraceptive continuously since >1 year and two women had an intrauterine device. The remaining women were tested during menses or the early follicular phase (day 1 to 10) of their menstrual cycle (*n *= 7).

### Experimental protocol

Parts of this experimental protocol, including dCA and IOH metrics from the group of eleven men included in this analysis have previously been published (Labrecque et al. 2017), as part of an investigation with a focus on the influence of CRF on dCA. Although this study employed the same experimental design, it represents a separate question (influence of sex on dCA) through the addition of a group of age and BMI matched women. Of note, inclusion (except for the sex of recruited participants) and exclusion criteria were the same between studies. As previously described (Labrecque et al. 2017), participants visited the laboratory on two occasions to perform: (1) an incremental cycling test for V̇O_2peak_ determination, and (2) anthropometrics, resting measurements and the evaluation of dCA and IOH. Participants were asked to avoid exercise training for at least 12 h, as well as alcohol and caffeine consumption for 24 h before each visit. All sessions and evaluations were executed in the exact same order for all participants and there was at least 48 h between testing sessions.

### Measurements

#### Systemic hemodynamics

Heart rate (HR) was measured using a 5‐lead ECG. Beat‐to‐beat BP and cardiac output (CO) were measured by the volume‐clamp method using a finger cuff (Nexfin, Edwards Lifesciences, Ontario, Canada). The cuff was placed on the right middle finger and referenced to the level of the heart using a height correct unit for BP correction. MAP was obtained by integration of the pressure curve divided by the duration of the cardiac cycle. The volume‐clamp method has been shown to reliably index the dynamic changes in beat‐to‐beat BP which correlate well with the intra‐arterial BP recordings and can be used to describe the dynamic relationship between BP and cerebral blood velocity (Sammons et al. 2007; Omboni et al. 1993).

#### Middle cerebral artery blood velocity

MCAv was monitored with a 2‐MHz pulsed transcranial Doppler ultrasound (Doppler Box; Compumedics DWL USA, Inc. San Juan Capistrano, CA). Identification and location of the left MCA was determined using standardized procedures (Willie et al. 2011). The probe was attached to a headset and secured with a custom‐made headband and adhesive conductive ultrasonic gel (Tensive, Parker Laboratory, Fairfield, NY, USA) to ensure a stable position and angle of the probe throughout testing.

#### End‐tidal partial pressure of carbon dioxide

End‐tidal partial pressure of carbon dioxide (P_ET_CO_2_) were measured during the baseline period before the beginning of the exercise protocol (in men only) or the sit‐to‐stand (in women only) and squat‐stand maneuvers (in both women and men) through a breath‐by‐breath gas analyzer (Breezesuite, MedGraphics Corp., MN) calibrated to known gas concentrations following manufacturer instructions before each evaluation.

#### Data acquisition

For each assessment, signals were analog‐to‐digital‐converted at 1kHz via an analog‐to‐digital converter (Powerlab 16/30 ML880; ADInstruments, Colorado Springs, CO, USA) and stored for subsequent analysis using commercially available software (LabChart version 7.1; ADInstruments).

### Visit 1

#### Peak oxygen consumption (V̇O_2peak_)

V̇O_2peak_ was determined during a progressive ramp exercise protocol performed on an electromagnetically braked upright cycle ergometer (Corival, Lode, the Netherlands). Following 3 min of rest, the evaluation started with a 1‐min warm‐up of unloaded pedaling followed by an incremental ramp protocol (from 22 to 30 W/min according to participant's history of training) to volitional exhaustion. Expired air was continuously recorded using a breath‐by‐breath gas analyzer (Breezesuite, MedGraphics Corp., MN, USA) for determination of V̇O_2peak_, carbon dioxide production (V̇CO_2_), respiratory exchange ratio (RER: V̇CO_2_/V̇O_2_), and P_ET_CO_2_. V̇O_2peak_ was defined as the highest 30‐sec averaged V̇O_2_, concurrent with a RER ≥ 1.15.

### Visit 2

#### Anthropometric measurements and resting hemodynamics

Height and body mass were measured in each participant. Resting hemodynamic measurements included MAP (volume‐clamp method using a finger cuff), which has been validated against intra‐arterial pressure (Labrecque et al. 2017) and CO (finger arterial pulse contour analysis), heart rate (HR; ECG), and mean MCAv (MCAv_mean_) (transcranial Doppler ultrasound), which were continuously monitored on a beat‐by‐beat basis during 5 min of seated rest. Cerebrovascular conductance index (CVCi; MCAv_mean_/MAP) and its reciprocal, resistance (CVRi; MAP/MCAv_mean_) was then calculated. P_ET_CO_2_ (breath‐by‐breath gas analyzer) was continuously monitored (in women) on a breath‐by‐breath basis. The average values of the last 15 sec of recording represented the baseline. Since P_ET_CO_2_ was measured only in women during this 5 min of seated rest for technical reasons, P_ET_CO_2_ values from the baseline period before the beginning of the exercise protocol (Visit 1) represented the baseline in men.

### Assessment of the dCA capacity and IOH

A multimetrics approach was employed to assess dCA to transient changes in MAP. We chose to force MAP oscillations using two separate techniques, to increase the input power (i.e., MAP) and improve the interpretation of the linear association between BP and MCAv (Smirl et al. 2015).

#### Sit‐to‐stand

Following 10 min of seated rest, participants rapidly (0–3 sec) stood up and maintained a standing position for 5 min without any movement or contraction of lower limb muscles. HR, CO, MAP, and MCAv_mean_ were continuously monitored during this evaluation of the sit‐to‐stand response. P_ET_CO_2_ was measured only in women for technical reasons. In addition to the characterization of dCA, this technique permitted us to focus on the initial phase of the orthostatic response within the first 15 sec after standing. In addition, we examined the prevalence of IOH, defined as a decrease in systolic BP ≥ 40 mmHg and/or a decrease in diastolic BP ≥ 20 mmHg during the first 15 sec of standing (Freeman et al. 2011).

#### Repeated squat‐stand maneuvers

Repeated squat‐stand maneuvers were performed after a minimum of 10 min of standing rest to ensure all cardiovascular variables had returned to baseline. Participants started in a standing position then squatted down until the back of their legs attained a ~ 90 degrees angle. This squat position was sustained for a specific time period, after which they moved to the standing position. Instructions were given and participants were asked to practice (2 or 3 squats) to ensure that they were squatting correctly. Then, participants performed 5‐min periods of repeated squat‐stand maneuvers at a frequency of 0.05 Hz (10‐sec squat, 10‐sec standing) and 0.10 Hz (5‐sec squat, 5‐sec standing) (Smirl et al. 2015). These large oscillations in MAP are extensively buffered by the cerebral vessels when executed at frequencies within the high‐pass filter buffering range (<0.20 Hz) (Zhang et al. 1998). The repeated squat‐stand maneuver optimizes the signal‐to‐noise ratio enhancing the reproducibility and interpretability of findings through a physiologically relevant MAP stimulus to the cerebrovasculature (Smirl et al. 2015). The sequence of the squat‐stand maneuvers was randomized between participants and each frequency was separated by 5 min of standing recovery after ensuring all cardiovascular variables returned to baseline. During these maneuvers, participants were instructed to maintain normal breathing and to avoid Valsalva maneuvers. The linear aspect of the dynamic MAP–MCAv relationship was characterized via TFA (see the “Data analysis and statistical approach” section). MAP, HR, MCAv_mean_, and P_ET_CO_2_ were continuously monitored during this evaluation. To evaluate whether squats induce changes in P_ET_CO_2_, an averaged P_ET_CO_2_ of the first and last five breaths of each maneuver (0.05 and 0.10 Hz) were calculated.

### dCA calculations

#### Acute cerebrovascular responses to hypotension induced by sit‐to‐stand

The following metrics were used to characterize the cerebral pressure–flow relationship to acute hypotension following the sit‐to‐stand: (1) the reduction in MAP and MCAv_mean_ to their respective nadir (absolute: ∆ MCAv_mean_, ∆ MAP; and relative to baseline: ∆ MCAv_mean_ (%), ∆ MAP (%)); (2) the percent reduction in MCAv_mean_ per percent reduction in MAP (%∆MCAv_mean_/%∆MAP); (3) the time delay before the onset of the regulatory change; (4) the rate of decline in MCAv_mean_ and; (5) the rate of regulation (RoR).

1. The reduction in MAP and MCAv_mean_ is the difference between baseline MAP or MCAv_mean_ (averaged over the last 15 sec of seated rest before standing) and minimum MAP or MCAv_mean_ recorded after the sit‐to‐stand.
2. %∆MCAv_mean_/%∆MAP upon standing was calculated as follows: [((baseline MCAv_mean_ – minimum MCAv_mean_)/baseline MCAv_mean_)/((baseline MAP – minimum MAP)/baseline MAP)].
3. The time delay before the onset of the regulatory change is the time lapse between the beginning of the sit‐to‐stand and the increase in CVCi (Labrecque et al. 2017). The onset of the regulatory response becomes visible when CVCi begins to continuously increase (without any subsequent transient reduction) during acute hypotension. This metric was assessed by two different observers (LL and PB).
4. The rate of decline in MCAv_mean_ upon standing was calculated as follows: [(%MCAv_mean_ at nadir − %MCAv_mean_ before decline)/(∆t)].
5. The physiological response to acute hypotension can be divided into two phases (Ogoh et al. 2008); Phase I is the time point after sit‐to‐stand where MCAv_mean_ changes are independent of any arterial baroreflex correction (1–7 sec after sit‐to‐stand (Deegan et al. 2009; Sorond et al. 2009; van Beek et al. 2008). Phase II is the time point starting at the onset of arterial baroreflex and continuing for 4 sec (Ogoh et al. 2008). During Phase I, the rate of change in CVCi is directly related to dCA, without arterial baroreflex regulation (Aaslid et al. 1989). RoR was calculated during Phase I using the following equation:

RoR=(ΔCVCi/Δt)/ΔMAP

where ∆CVCi/∆*t* is the linear regression slope between CVCi and time (*t*) during Phase I (a 2.5‐sec interval (∆*t*) after individually determined onset of the regulatory change following sit‐to‐stand was used for the analysis of RoR), and ∆MAP is calculated by subtracting baseline MAP from averaged MAP during Phase I (Ogoh et al. 2008; Aaslid et al. 1989).

#### Assessment of the dynamic relationship between MAP and MCAv

Data were analyzed using the commercially available software Ensemble (Version 1.0.0.14, Elucimed, Wellington, New Zealand) and are in accordance with the recommendations of the Cerebral Autoregulation Research Network (CARNet) (Claassen et al. 2016). Beat‐to‐beat MAP and MCAv signals were spline interpolated and re‐sampled at 4 Hz for spectral analysis and TFA based on the Welch algorithm. Each 5‐min recording was first subdivided into five successive windows that overlapped by 50%. Data within each window were linearly detrended and passed through a Hanning window prior to discrete Fourier transform analysis. For TFA, the cross‐spectrum between MAP and MCAv was determined and divided by the MAP auto‐spectrum to derive the transfer function coherence (fraction of the MAP which is linearly related to MCAv), absolute gain (cm/sec/mmHg) (amplitude of MCAv change for a given oscillation in MAP), normalized gain (%/mmHg), and phase (radians) (difference of the timing of the MAP and MCAv waveforms).

TFA coherence, gain, and phase of the forced MAP oscillations were sampled at the point estimate of the driven frequency (0.05 and 0.10 Hz). These point estimates were selected as they are in the very low (0.02–0.07 Hz) and low (0.07–0.20 Hz) frequency ranges where dCA is thought to be most operant (Smirl et al. 2015). Only the TFA phase and gain values where coherence exceeded 0.50 were included in analysis to ensure the measures were robust for subsequent analysis (Zhang et al. 1998). Phase wrap‐around was not present when coherence exceed 0.50 at any of the point estimate values for squat‐stand maneuvers.

### Statistical analysis

Following the confirmation of normal distribution of data using Shapiro–Wilk normality tests, between‐group differences were analyzed using independent samples t‐tests. Difference in the prevalence of IOH between groups was analyzed using the Fisher's exact test. Relationships between variables were determined using Pearson product‐moment. Statistical significance was established a priori at *P < *0.05 for all two‐tailed tests. Data are expressed as mean ± standard deviation.

## Results

### Dropout/Compliance

Three participants (two women and one men) were excluded from the sit‐to‐stand analysis because of an insufficient reduction in MAP (<10 mmHg) (Subudhi et al. 2015). The final sample size for the responses to acute hypotension following the sit‐to‐stand was nine women and 10 men. Four participants were excluded from the TFA because of an inconsistent BP trace and a premature ending of the repeated squat‐stand maneuvers related to the appearance of orthostatic symptoms (two women) or the absence of an appropriate ECG signal (two men). The final sample size for the TFA of forced oscillations in MAP and MCAv was nine women and nine men.

### Participant characteristics and baseline systemic and cerebrovascular hemodynamics

Age and BMI were comparable between groups. Volume of weekly training was also similar in both groups. Body mass, height, P_ET_CO_2_, and V̇O_2peak_ were lower in women (Table 1). Baseline MCAv_mean_ (+14 cm sec^−1^; *P < *0.001) and CVCi (+0.14 cm·sec^−1^·mmHg^−1^; *P = *0.002) were higher, whereas CVRi was lower (−0.29 mmHg·cm·sec^−1^ *P = *0.001), in women. All other baseline systemic hemodynamics were similar between groups (Table 1).

**Table 1:** Baseline characteristics and resting values between women and men

	Women	Men	*P*‐values
N	11	11	
Baseline characteristics			
Age (years)	25 ± 4	24 ± 2	0.610
Height (m)	1.64 ± 0.07	1.78 ± 0.09	<0.001
Body mass (kg)	61.0 ± 5.7	72.5 ± 10.4	0.007
Body mass index (kg/m^2^)	23 ± 2	23 ± 2	0.992
Training volume (min)	466 ± 151	511 ± 188	0.548
Peak oxygen uptake (mL/kg·min^−1^)	48.1 ± 4.1	56.8 ± 4.4	<0.001
Resting values			
Heart rate (bpm)	73 ± 11	74 ± 12 (n=10)	0.853
Cardiac output (L/min)	5.6 ± 1.2	6.7 ± 0.9	0.250
Mean arterial pressure (mmHg)	96 ± 10	97 ± 9	0.919
Middle cerebral artery mean blood velocity (cm·sec^−1^)	75 ± 7	61 ± 7	<0.001
Cerebrovascular resistance index (mmHg·cm·sec^−1^)	1.30 ± 0.16	1.59 ± 0.20	0.001
Cerebrovascular conductance index (cm·sec^−1^·mmHg^−1^)	0.78 ± 0.10	0.64 ± 0.08	0.002
End‐tidal partial pressure of carbon dioxide (mmHg)	36.9 ± 2.3	42.5 ± 3.5	<0.001

Data are presented as mean ± SD.

### Influence of sex on dCA

#### Responses to acute hypotension following the sit‐to‐stand

**Figure 1:**
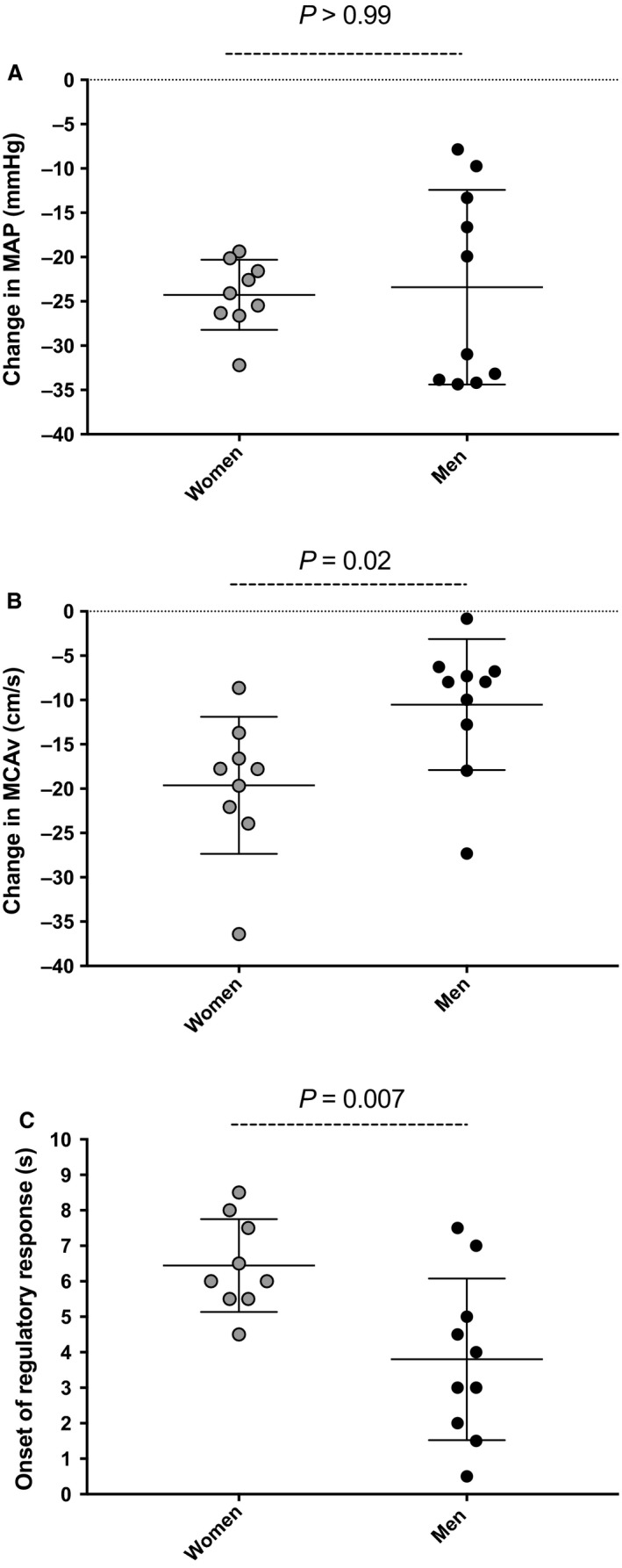
Cerebrovascular responses following sit‐to‐stand. Change in mean arterial pressure (MAP) from baseline (A), change in middle cerebral artery mean blood velocity (MCAv_mean_) from baseline (B), and onset of the regulatory response (C). Shaded circles indicate women and black circles, men.

MAP (96 ± 10 vs. 97 ± 9 mmHg; *P = *0.919) was comparable between groups and MCAv_mean_ (75 ± 7 vs. 61 ± 7 cm sec^−1^; *P = *0.0001) was higher in women than men at baseline. Upon standing, although the reduction [absolute (Fig. 1) and relative (−26 ± 4 vs. −25 ± 11%; *P = *0.862)] in MAP to nadir was similar between women and men, the absolute decrease in MCAv_mean_ was of greater amplitude in women (−20 ± 8 vs. −11 ± 7 cm sec^−1^; *P = *0.018), whereas the relative decrease in MCAv_mean_ tended to be greater (−26 ± 9 vs. −17 ± 11%; *P* = 0.061). However, the rate of decline in MCAV_mean_ was similar between groups (−4.93 ± 1.87 vs. −5.88 ± 2.84% sec^−1^; *P* = 0.37). There were no group differences in the time taken to reach the nadir for MAP (7 ± 1 vs. 7 ± 2 sec; *P = *0.510). Although peak HR upon standing was similar between groups (100 ± 10 vs. 93 ± 11 bpm; *P* = 0.41), peak CO was significantly lower in women vs. men (8.6 ± 1.6 vs. 10.8 ± 2.0 L/min; *P* = 0.005). The time delay before the onset of the regulatory change was almost two‐fold longer in women (*P = *0.007; Fig. 1) following the sit‐to‐stand. After the onset of the regulatory response, RoR was not different between groups (0.26 ± 0.27 vs. 0.23 ± 0.36 sec^−1^; *P* = 0.867). In women, mean change in P_ET_CO_2_ from baseline to average MAP during the first 15 sec following the sit‐to‐stand was −0.8 ± 1.1 mmHg.

#### TFA of forced oscillations in MAP and MCAv

**Figure 2:**
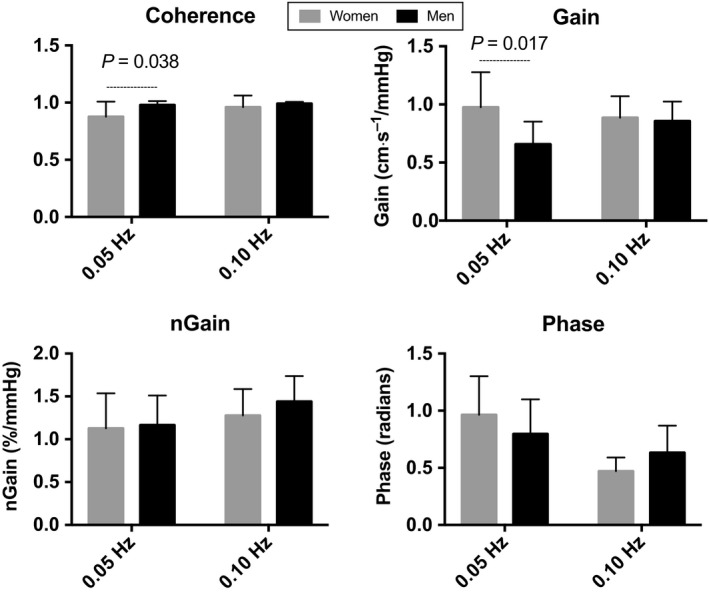
Transfer function analysis of forced oscillation in mean arterial pressure and middle cerebral artery blood velocity. Group averaged coherence, gain, normalized gain (nGain) and phase for 0.05 and 0.10 Hz squat‐stand.

MAP and MCAv power spectrum densities (0.05 and 0.10 Hz) during forced oscillations were not different between women and men (Table 2). Coherence during 0.05 Hz squat‐stand was lower in women than men (*P = *0.038). Absolute TFA gain during 0.05 Hz squat‐stand was higher in women compared to men (*P = *0.017). All the other metrics were not different between women and men (Table 2 and Fig. 2). Changes in P_ET_CO_2_ from the beginning of each squat‐stand maneuver (0.05 Hz: +1.6 ± 1.2 vs. +1.4 ± 2.3 mmHg; *P = *0.50 and 0.10 Hz: +1.3 ± 0.7 vs. +2.0 ± 3.2 mmHg *P = *0.66) were comparable between women and men.

**Table 2:** Power spectrum densities of forced oscillations in mean arterial pressure and middle cerebral artery blood velocity during squat‐stand maneuvers

	Women	Men	*P*‐values
0.05 Hz squat‐stand			
*N*	9	9	
Mean arterial pressure power (mmHg^2^)	8517 ± 3424	11,599 ± 7097	0.220
Middle cerebral artery blood velocity power (cm/s^2^)	6504 ± 4161	7957 ± 3859	0.454
0.10 Hz squat‐stand			
*N*	10	9	
Mean arterial pressure power (mmHg^2^)	2491 ± 2539	11,649 ± 13,294	0.059
Middle cerebral artery blood velocity power (cm/s^2^)	2180 ± 4321	5472 ± 2107	0.216

Values are mean ± SD.

### Initial orthostatic hypotension

The prevalence of IOH (women: 4/9 vs. men: 5/9, *P = *0.637) was not different between groups and syncope‐related symptoms were not reported by the participants who experienced IOH. There were no correlations between metrics of dCA and decreases in MAP and MCAv_mean_ to their nadir upon standing (data not shown).

## Discussion

The main findings of this study were threefold: young fit women had 1) a delayed onset of their cerebral autoregulatory response; 2) a greater decrease in MCAv_mean_ in response to transient hypotension induced by a sit‐to‐stand; and 3) higher absolute TFA gain during 0.05 Hz repeated squat‐stand maneuvers. Taken together, these findings imply that the brain vasculature of these young fit women has a reduced ability to dampen fast and large MAP oscillations compared to men. Finally, the prevalence of IOH was similar in women and men, and was not associated with dCA metrics in our women. Overall, these findings support the notion that despite differences in dCA between young fit women and men, this was not related to symptoms of cerebral hypoperfusion during orthostasis.

### Resting cerebral hemodynamics

In healthy young adults, resting CBF has often been reported to be higher in women compared to age‐matched men (Edgell et al. 2012; Marinoni et al. 1998; Tegeler et al. 2013; Liu et al. 2016). It has been speculated this CBF, when monitored using transcranial (intra‐cranial arteries) or duplex Doppler (extra‐cranial arteries) ultrasound, is associated with circulating ovarian hormones and the menstrual cycle (Brackley et al. 1999; Krejza et al. 2001, 2003). Specifically, blood flow through the carotid arteries increases throughout the follicular phase and reaches its maximum at day 14, whereas cerebrovascular resistances in the MCA are smaller than the luteal phase (Brackley et al. 1999; Krejza et al. 2001). The current results of greater resting MCAv_mean_ and CVCi, and lower CVRi, in women are in agreement with these findings (Table 1).

### dCA

#### Acute hypotension induced by sit‐to‐stand

The literature related to the influence of sex on the cerebrovascular response to BP changes is sparse and limited to just a few variables. To the best of the authors knowledge currently only one study has shown differential regulation between women and men in response to a sit‐to‐stand maneuver (Deegan et al. 2011). Deegan et al. showed that women had improved dCA metrics with higher autoregulation index (ARI), smaller reductions in MAP and MCAv_mean_ as well as a lower reduction in %∆MCAv_mean_/%∆MAP. However, subjects were aged over 70 years old, so the potential confounding effects of circulating sex hormones could be discounted (Deegan et al. 2011). In younger individuals, men seem to possess a better static CA during head‐up tilt (Wang et al. 2010) and higher cerebrovascular resistance while standing than women (Abidi et al. 2017). Conversely, others reported no sex differences in CBF regulation during head‐up tilt (Hazlett and Edgell 2018) or the sit‐to‐stand maneuver (Edgell et al. 2012).

**Figure 3:**
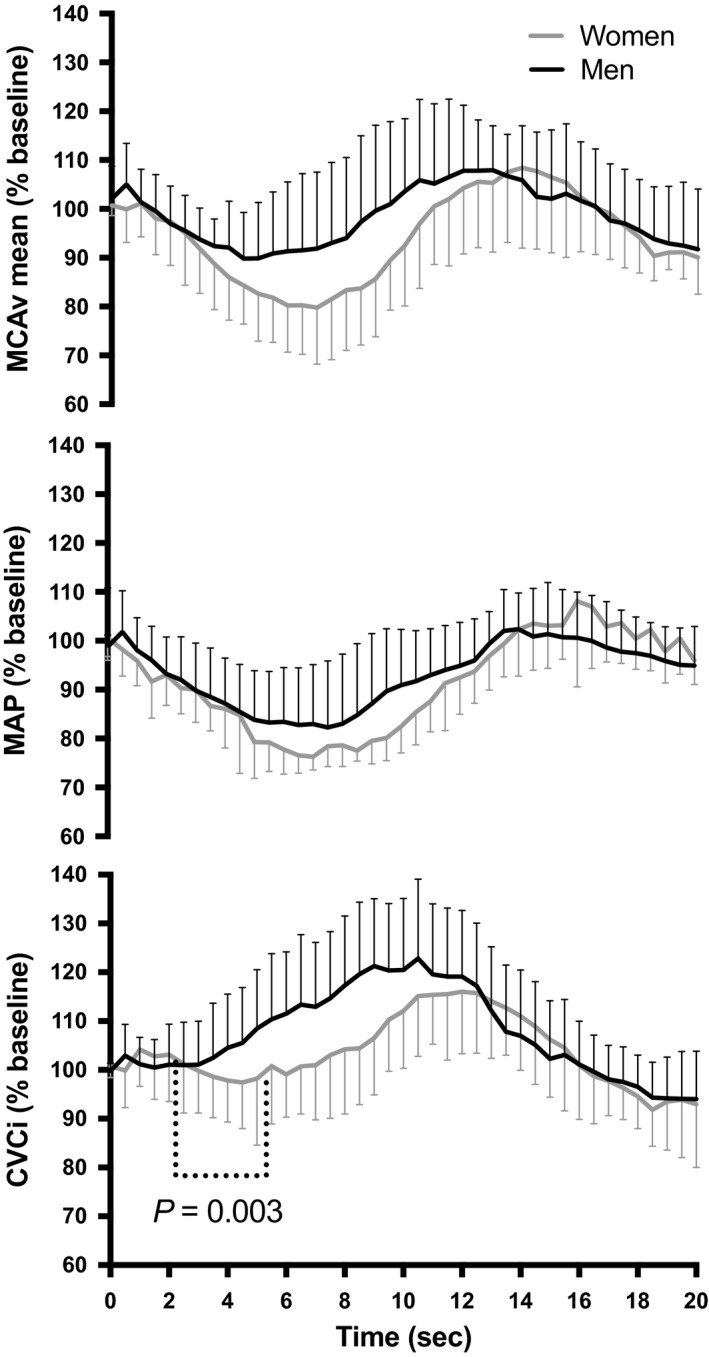
Normalized response of mean arterial pressure (MAP), middle cerebral artery mean blood velocity (MCAv_mean_) and cerebrovascular conductance index (CVCi) in women (gray line) and men (black line) following sit‐to‐stand. Time 0 indicates the transition from sitting to standing.

In this study, we demonstrate an impairment in the cerebrovasculature of young fit women when this system is challenged via a large and rapid reductions in BP. Despite a comparable MAP reduction between groups, women had a delayed onset of the regulatory response, as well as a larger reduction in MCAv_mean_ following sit‐to‐stand (Figs. 1, 3). Differences in analytical techniques might explain disparities between the current results and those from the broader literature. In fact, in two studies where a sit‐to‐stand was included (Abidi et al. 2017; Edgell et al. 2012) the sitting and standing positions were compared during the steady‐state phases (in contrast to the current design which assessed the dynamic phase) associated with the posture change. By comparing only the averaged data of the last minute at each position in their healthy men and women (5 min seated and 10 min standing) (Abidi et al. 2017), variables (i.e., HR, MAP, MCAv_mean_, cerebrovascular resistance) will have had sufficient time for the baroreceptors to have adjusted and enabled recovery from the acute hypotension, thus reducing the acute influence of the orthostatic challenge on the metrics of interest. The discrepancy between the current findings and the previous literature further emphasizes the importance of analyzing the dynamic response of the sit‐to‐stand (i.e., dCA) instead of simply comparing steady‐state hemodynamics before and after a given BP challenge (i.e., static CA). Therefore, it is important to consider the influence of various measures during both static and dynamic cerebral autoregulatory challenges (Tzeng et al. 2012) (such as %ΔMCAv_mean_/%ΔMAP, the onset of the regulatory response and RoR) when assessing sex differences in the CBF response to a sit‐to‐stand. Also, changes in P_ET_CO_2_ during the first 15 sec upon standing were minimal in women in this study. Although this variable could not be measured in men for technical reasons, sex does not seem to influence the change in P_ET_CO_2_ during a sit‐to‐stand, at least in an older population (Deegan et al. 2009).

#### TFA of spontaneous and forced oscillations

The literature examining the influence of sex on TFA of spontaneous and forced oscillations is limited. To the best of the authors knowledge, only two studies have used TFA to examine sex differences in dCA. In a population ranging in age from 21 to 80 years old, Xing et al. (2017) reported lower VLF gain and higher phase during 0.05 Hz repeated squat‐stand maneuvers as compared with men, again suggesting a better dCA in women. In contrast to this investigation, Patel et al. found no differences in dCA between sexes using TFA of spontaneous oscillations in 129 participants with a mean age of ~57 years (Patel et al. 2016).

This study revealed young fit women had a higher gain during 0.05 Hz repeated squat‐stand maneuvers (Fig. 2) indicative of greater change in MCAv for a given change in MAP compared to men. This higher gain during 0.05 Hz repeated squat‐stand maneuvers also relates to the lower MAP power spectrum densities at 0.05 Hz. Indeed, the trend toward lower MAP power spectrum densities in women with a similar MCAv power spectrum densities between sexes during 0.05 Hz squat‐stand would indicate more of the BP being transferred to the women brain. Discrepancies between the current results and the previous literature might come from the age differences between our studied populations. Many of the women included in the previous studies were probably postmenopausal, which could attenuate the influence of hormone differences in dCA between sexes (Deegan et al. 2011). Consistent with this notion are previous reports which demonstrated there were no sex differences in cerebral hemodynamics between old women and young men (Edgell et al. 2012; Liu et al. 2016). Further research is needed to better understand the influence of the menstrual cycle on TFA metrics. Of note, P_ET_CO_2_ is unlikely to explain the reported differences in dCA metrics gathered during forced oscillations in BP considering that small changes in P_ET_CO_2_ during repeated squat‐stand maneuvers were not different between women and men.

#### To what extent are the differences in dCA in healthy active women physiologically/clinically meaningful?

In this study, the prevalence of IOH was similar between women and men, even though women showed a diminished dCA and a greater reduction in MCAv_mean_ upon standing (Figs. 1, 2). However, the current results did not correlate dCA metrics and reductions in MAP and MCAv_mean_ during orthostatic stress induced by the sit‐to‐stand in women. These results suggest the subtle changes in dCA in young fit women do not translate into functional outcome, at least in response to a sit‐to‐stand. Hypothetically, a delayed initiation of autoregulation in response to a rapid reduction in BP may extend the time period associated with pressure‐passive cerebral blood velocity without counterregulation. Our findings thus bring into question the physiological validity of the subtle dCA differences which have been observed in otherwise healthy young women. These dCA changes may simply not be important enough to influence the prevalence of IOH, as the alterations in both the sit‐to‐stand and TFA data indicate the cerebrovasculature of the women may be more compliant and be able to better withstand alterations in BP induced in the current investigation. Larger reductions in MAP may thus be necessary to induce orthostatic symptoms in the presence of functional impairments in dCA. The absence of associations between the attenuated dCA and the prevalence of IOH described here could be related to a higher resting CBF, as reported by others (Abidi et al. 2017; Edgell et al. 2012; Marinoni et al. 1998), allowing women to better face a rapid transient hypotension induced by a sit‐to‐stand compared to men. These dCA changes could then become clinically meaningful with a larger BP reduction.

### Limitations

Some limitations to our study deserve further discussion. Only young healthy and fit women and men participated to this study and the results cannot be generalized to other populations (such as older individuals or hypertensive patients). Furthermore, since orthostatic tolerance has been associated with the posterior circulation, it would have been interesting to measure blood velocity in the posterior cerebral artery (Kay and Rickards 2016).

P_ET_CO_2_ was not available in men during the sit‐to‐stand. Baseline P_ET_CO_2_ in men was thus determined from the baseline period before the beginning of the exercise protocol. Although this could have partly explained the difference in baseline P_ET_CO_2_ between groups, which supports the literature (Dhokalia et al. 1998), women had comparable P_ET_CO_2_ values during the baseline period before the beginning of the exercise protocol (Visit 1) and the 5 min of baseline rest (Visit 2) (36.9 ± 2.3 vs. 36.5 ± 2.8 mmHg *P = *0.47*)*. Men most likely had similar P_ET_CO_2_ values between visits as well. In addition, changes in P_ET_CO_2_ during this maneuver was minimal in women and sex does not seem to influence the change in P_ET_CO_2_ during a sit‐to‐stand in an older population (Deegan et al. 2009). This variable most likely played a minimal role in the metrics related to the sit‐to‐stand. Indeed, since women had lower P_ET_CO_2_ at baseline (which would lead to vasoconstriction), it did not play a role in the augmented gain values for either the sit‐to‐stand nor TFA parameters. A reduction in CO_2_ should cause a decrease in gain and this was not observed. Instead, the altered CO_2_ at baseline likely did not cause the noted differences in the study and in fact may have instead limited the extent of them.

Further to this point, cerebral blood velocity in the MCA was measured with transcranial Doppler ultrasound and is representative of flow if the diameter of the arteries remains constant. Changes in MAP and P_ET_CO_2_ have been associated with changes in the diameter of the internal carotid artery and MCA. However, the physiological range of variation in MAP and P_ET_CO_2_ from this study will most likely be associated with a minor effect on the diameter of the MCA (Verbree et al. 2014; Lewis et al. 2015). Participants performed only one sit‐to‐stand. Further research, where systemic and cerebral hemodynamics responses to several sit‐to‐stand maneuvers are averaged, will be necessary to support findings from this study.

Considering the women in the current investigation included: a group of women taking oral contraceptives continuously (*n *= 2), having an intrauterine device (*n *= 2) or were assessed during days 1–10 of their menstrual cycle (*n *= 7), we are unable to ascertain if the elevated CBF in the current investigation was influenced by the oscillatory nature of these hormones throughout the menstrual cycle. Further research is warranted to determine the specific effects the stages of the menstrual cycle play on these measures. In addition, we cannot rule out the possibility that dCA changes reported in this study could be amplified and ultimately affect the incidence of IOH later in the menstrual cycle. Indeed, presyncopal symptoms are more frequent during the menses phase of the menstrual cycle (Muppa et al. 2013). Menses usually last from day 0 to 5. However, some of our women were tested after day 5. Therefore, their orthostatic response could be better following the menses phase leading to a lower prevalence of orthostatic hypotension and less symptoms of cerebral hypoperfusion in our cohort. Of note, Abidi et al. recently assessed static CA and peripheral hemodynamics during a sit‐to‐stand protocol in oral contraceptives and nonoral contraceptives users, as well as during the high and low hormones phases. They reported differences in MAP regulation, but no impact of the menstrual cycle or oral contraceptives use on the cerebrovascular response (Abidi et al. 2017).

Although comparable in terms of previous weekly training volume, women and men were not matched for V̇O_2peak_ and it could have influenced our results. We have recently reported a reduced dCA with elevated CRF when the brain is challenged with large and rapid MAP change in men (Labrecque et al. 2017). We speculate the differences in dCA metrics observed between women and men in this study would be even more important if women had a higher CRF to match men's level. However, since CRF is usually superior in men versus women for a similar charge of training, a matching of V̇O_2peak_ between male and female athletes would not represent a real‐life situation (Cureton 1981). Either women would be too trained or men untrained.

## Conclusions

These results indicate the cerebrovasculature of young fit women has an attenuated ability to react to changes in BP compared to men, when the brain is challenged with large and rapid BP oscillations. However, these subtle dCA changes are not translated into functional impairments using initial orthostatic hypotension as the functional outcome, which could be related to a higher resting cerebral blood flow, allowing women to better face a rapid transient hypotension induced by a sit‐to‐stand compared to men.

## Conflict of Interests

The authors declare that there is no conflict of interest.

## References

[phy213984-bib-0001] Aanerud, J. , P. Borghammer , A. Rodell , K. Y. Jonsdottir , and A. Gjedde . 2017 Sex differences of human cortical blood flow and energy metabolism. J. Cereb. Blood Flow Metab. 37:2433–2440. 27629099 10.1177/0271678X16668536 PMC5531342

[phy213984-bib-0002] Aaslid, R. , K. F. Lindegaard , W. Sorteberg , and H. Nornes . 1989 Cerebral autoregulation dynamics in humans. Stroke 20:45–52. 2492126 10.1161/01.str.20.1.45

[phy213984-bib-0003] Abidi, S. , M. Nili , S. Serna , S. Kim , C. Hazlett , and H. Edgell . 2017 Influence of sex, menstrual cycle, and oral contraceptives on cerebrovascular resistance and cardiorespiratory function during Valsalva or standing. J. Appl. Physiol. 123:375–386. 28522756 10.1152/japplphysiol.00035.2017 PMC5583611

[phy213984-bib-0004] Ali, Y. S. , N. Daamen , G. Jacob , J. Jordan , J. R. Shannon , I. Biaggioni , et al. 2000 Orthostatic intolerance: a disorder of young women. Obstet. Gynecol. Surv. 55:251–259. 10758621 10.1097/00006254-200004000-00025

[phy213984-bib-0005] Brackley, K. J. , M. M. Ramsay , F. Broughton Pipkin , and P. C. Rubin . 1999 The effect of the menstrual cycle on human cerebral blood flow: studies using Doppler ultrasound. Ultrasound Obstet. Gynecol. 14:52–57. 10461339 10.1046/j.1469-0705.1999.14010052.x

[phy213984-bib-0006] Claassen, J. A. H. R. , B. D. Levine , and R. Zhang . 2009 Dynamic cerebral autoregulation during repeated squat‐stand maneuvers. J. Appl. Physiol. 106:153–160. 18974368 10.1152/japplphysiol.90822.2008 PMC2636935

[phy213984-bib-0007] Claassen, J. A. H. R. , vanMeel‐ den Abeelen A. S. S. , D. M. Simpson , and R. B. Panerai , International Cerebral Autoregulation Research Network (CARNet) . 2016 Transfer function analysis of dynamic cerebral autoregulation: a white paper from the International Cerebral Autoregulation Research Network. J. Cereb. Blood Flow Metab. 36: 665–680. 26782760 10.1177/0271678X15626425 PMC4821028

[phy213984-bib-0008] Cureton, K. J. 1981 Matching of male and female subjects using VO_2_ max. Res. Q. Exerc. Sport 52:264–268. 7268184 10.1080/02701367.1981.10607865

[phy213984-bib-0009] Deegan, B. M. , F. A. Sorond , A. Galica , L. A. Lipsitz , G. ÓLaighin , and J. M. Serrador . 2011 Elderly women regulate brain blood flow better than men do. Stroke 42: 1988–1993. 21566238 10.1161/STROKEAHA.110.605618 PMC7111558

[phy213984-bib-0010] Deegan, B. M. , F. A. Sorond , L. A. Lipsitz , G. ÓLaighin , and J. M. Serrador . 2009 Gender related differences in cerebral autoregulation in older healthy subjects. Conf. Proc. IEEE Eng. Med. Biol. Soc. 2009: 2859–2862. 19964277 10.1109/IEMBS.2009.5333604 PMC2915823

[phy213984-bib-0011] Dhokalia, A. , D. J. Parsons , and D. E. Anderson . 1998 Resting end‐tidal CO_2_ association with age, gender, and personality. Psychosom. Med. 60:33–37. 9492236 10.1097/00006842-199801000-00007

[phy213984-bib-0012] Edgell, H. , A. D. Robertson , and R. L. Hughson . 2012 Hemodynamics and brain blood flow during posture change in younger women and postmenopausal women compared with age‐matched men. J. Appl. Physiol. 112:1482–1493. 22362404 10.1152/japplphysiol.01204.2011

[phy213984-bib-0013] Freeman, R. , W. Wieling , F. B. Axelrod , D. G. Benditt , E. Benarroch , I. Biaggioni , et al. 2011 Consensus statement on the definition of orthostatic hypotension, neurally mediated syncope and the postural tachycardia syndrome. Clin. Auton. Res. 21:69–72. 21431947 10.1007/s10286-011-0119-5

[phy213984-bib-0014] Fu, Q. , A. Arbab‐Zadeh , M. A. Perhonen , R. Zhang , J. H. Zuckerman , and B. D. Levine . 2004 Hemodynamics of orthostatic intolerance: implications for gender differences. AJP: Heart Circ. Physiol. 286: H449–57. 10.1152/ajpheart.00735.2002 14527942

[phy213984-bib-0015] Hazlett, C. , and H. Edgell . 2018 Chemoreflex function and brain blood flow during upright posture in men and women. Physiol. Rep. 6: e13571. 10.14814/phy2.13571 PMC5789659 29333725

[phy213984-bib-0016] Kastrup, A. , C. Thomas , C. Hartmann , and M. Schabet . 1997 Sex dependency of cerebrovascular CO_2_ reactivity in normal subjects. Stroke 28:2353–2356. 9412613 10.1161/01.str.28.12.2353

[phy213984-bib-0981] Kay, V. L. , and C. A. Rickards . 2016 The role of cerebral oxygenation and regional cerebral blood flow on tolerance to central hypovolemia. Am. J. Physiol. Regul. Integr. Comp. Physiol. 310:R375‐83. 26676249 10.1152/ajpregu.00367.2015

[phy213984-bib-0017] Krejza, J. , Z. Mariak , M. Huba , S. Wolczynski , and J. Lewko . 2001 Effect of endogenous estrogen on blood flow through carotid arteries. Stroke 32:30–36. 11136910 10.1161/01.str.32.1.30

[phy213984-bib-0018] Krejza, J. , J. Siemkowicz , M. Sawicka , A. Szylak , J. Kochanowicz , Z. Mariak , et al. 2003 Oscillations of cerebrovascular resistance throughout the menstrual cycle in healthy women. Ultrasound Obstet. Gynecol. 22:627–632. 14689537 10.1002/uog.907

[phy213984-bib-0019] Labrecque, L. , K. Rahimaly , S. Imhoff , M. Paquette , O. Le Blanc , S. Malenfant , et al. 2017 Diminished dynamic cerebral autoregulatory capacity with forced oscillations in mean arterial pressure with elevated cardiorespiratory fitness. Physiol. Rep. 5: e13486. 10.14814/phy2.13486 PMC5688778 29122957

[phy213984-bib-0020] Lewis, N. C. S. , K. J. Smith , A. R. Bain , K. W. Wildfong , T. Numan , and P. N. Ainslie . 2015 Impact of transient hypotension on regional cerebral blood flow in humans. Clin. Sci. 129:169–178. 25697830 10.1042/CS20140751

[phy213984-bib-0021] Liu, W. , X. Lou , and L. Ma . 2016 Use of 3D pseudo‐continuous arterial spin labeling to characterize sex and age differences in cerebral blood flow. Neuroradiology 58:943–948. 27380039 10.1007/s00234-016-1713-y

[phy213984-bib-0022] Marinoni, M. , A. Ginanneschi , D. Inzitari , S. Mugnai , and L. Amaducci . 1998 Sex‐related differences in human cerebral hemodynamics. Acta Neurol. Scand. 97:324–327. 9613563 10.1111/j.1600-0404.1998.tb05961.x

[phy213984-bib-0023] Muppa, P. , R. S. Sheldon , M. McRae , N. R. Keller , D. Ritchie , A. D. Krahn , et al. 2013 Gynecological and menstrual disorders in women with vasovagal syncope. Clin. Auton. Res. 23:117–122. 23467969 10.1007/s10286-013-0190-1 PMC3681885

[phy213984-bib-0982] Ogoh, S. , R. M. Brothers , W. L. Eubank , and P. B. Raven . 2008 Autonomic neural control of the cerebral vasculature: acute hypotension. Stroke 39:1979–1987. 18451346 10.1161/STROKEAHA.107.510008

[phy213984-bib-0024] Omboni, S. , G. Parati , A. Frattola , E. Mutti , M. Di Rienzo , P. Castiglioni , et al. 1993 Spectral and sequence analysis of finger blood pressure variability. Comparison with analysis of intra‐arterial recordings. Hypertension 22: 26–33. 8319990 10.1161/01.hyp.22.1.26

[phy213984-bib-0025] Patel, N. , R. B. Panerai , V. Haunton , E. Katsogridakis , N. P. Saeed , A. Salinet , et al. 2016 The Leicester cerebral haemodynamics database: normative values and the influence of age and sex. Physiol. Meas. 37:1485–1498. 27511128 10.1088/0967-3334/37/9/1485

[phy213984-bib-0026] Romme, J. J. C. M. , N. van Dijk , K. R. Boer , L. R. C. Dekker , J. Stam , J. B. Reitsma , et al. 2008 Influence of age and gender on the occurrence and presentation of reflex syncope. Clin. Auton. Res. 18:127–133. 18449594 10.1007/s10286-008-0465-0

[phy213984-bib-0027] Sammons, E. L. , N. J. Samani , S. M. Smith , W. E. Rathbone , S. Bentley , J. F. Potter , et al. 2007 Influence of noninvasive peripheral arterial blood pressure measurements on assessment of dynamic cerebral autoregulation. J. Appl. Physiol. 103:369–375. 17463300 10.1152/japplphysiol.00271.2007

[phy213984-bib-0028] Simpson, D. , and J. Claassen . 2018 CrossTalk opposing view: dynamic cerebral autoregulation should be quantified using induced (rather than spontaneous) blood pressure fluctuations. J. Physiol. 596:7–9. 29207208 10.1113/JP273900 PMC5746528

[phy213984-bib-0029] Smirl, J. D. , K. Hoffman , Y.‐C. Tzeng , A. Hansen , and P. N. Ainslie . 2015 Methodological comparison of active‐ and passive‐driven oscillations in blood pressure; implications for the assessment of cerebral pressure‐flow relationships. J. Appl. Physiol. 119:487–501. 26183476 10.1152/japplphysiol.00264.2015 PMC4556839

[phy213984-bib-0984] Sorond, F. A. , J. M. Serrador , R. N. Jones , M. L. Shaffer , and L. A. Lipsitz . 2009 The sit‐to‐stand technique for the measurement of dynamic cerebral autoregulation. Ultrasound Med. Biol. 35:21–29. 18834658 10.1016/j.ultrasmedbio.2008.08.001 PMC2680703

[phy213984-bib-0030] Subudhi, A. W. , K. Grajzel , R. J. Langolf , R. C. Roach , R. B. Panerai , and J. E. Davis . 2015 Cerebral autoregulation index at high altitude assessed by thigh‐cuff and transfer function analysis techniques. Exp. Physiol. 100:173–181. 25480158 10.1113/expphysiol.2014.082479

[phy213984-bib-0031] Tegeler, C. H. , K. Crutchfield , M. Katsnelson , J. Kim , R. Tang , L. Passmore Griffin , et al. 2013 Transcranial Doppler velocities in a large, healthy population. J. Neuroimaging 23:466–472. 23157483 10.1111/j.1552-6569.2012.00711.x

[phy213984-bib-0032] Tzeng, Y.‐C. , P. N. Ainslie , W. H. Cooke , K. C. Peebles , C. K. Willie , B. A. MacRae , et al. 2012 Assessment of cerebral autoregulation: the quandary of quantification. AJP: Heart Circ. Physiol. 303:H658–H671. 10.1152/ajpheart.00328.2012 22821992

[phy213984-bib-0033] Tzeng, Y.‐C. , and P. N. Ainslie . 2014 Blood pressure regulation IX: cerebral autoregulation under blood pressure challenges. Eur. J. Appl. Physiol. 114:545–559. 23737006 10.1007/s00421-013-2667-y PMC3929776

[phy213984-bib-0983] van Beek, A. H. , J. A. Claassen , M. G. Rikkert , and R. W. Jansen . 2008 Cerebral autoregulation: an overview of current concepts and methodology with special focus on the elderly. J. Cereb. Blood Flow Metab. 28:1071–1085. 18349877 10.1038/jcbfm.2008.13

[phy213984-bib-0034] Verbree, J. , A.‐S. G. T. Bronzwaer , E. Ghariq , M. J. Versluis , M. J. A. P. Daemen , van Buchem M. A. , et al. 2014 Assessment of middle cerebral artery diameter during hypocapnia and hypercapnia in humans using ultra‐high‐field MRI. J. Appl. Physiol. 117:1084–1089. 25190741 10.1152/japplphysiol.00651.2014

[phy213984-bib-0035] Wang, Y.‐J. , A.‐C. Chao , C.‐P. Chung , Y.‐J. Huang , and H.‐H. Hu . 2010 Different cerebral hemodynamic responses between sexes and various vessels in orthostatic stress tests. J. Ultrasound Med. 29:1299–1304. 20733185 10.7863/jum.2010.29.9.1299

[phy213984-bib-0036] Willie, C. K. , F. L. Colino , D. M. Bailey , Y.‐C. Tzeng , G. Binsted , L. W. Jones , et al. 2011 Utility of transcranial Doppler ultrasound for the integrative assessment of cerebrovascular function. J. Neurosci. Methods 196:221–237. 21276818 10.1016/j.jneumeth.2011.01.011

[phy213984-bib-0037] Xing, C.‐Y. , T. Tarumi , R. L. Meijers , M. Turner , J. Repshas , L. Xiong , et al. 2017 Arterial pressure, heart rate, and cerebral hemodynamics across the adult life span. Hypertension 69:712–720. 28193707 10.1161/HYPERTENSIONAHA.116.08986 PMC5344744

[phy213984-bib-0038] Zhang, R. , J. H. Zuckerman , C. A. Giller , and B. D. Levine . 1998 Transfer function analysis of dynamic cerebral autoregulation in humans. Am. J. Physiol. 274:H233–H241. 9458872 10.1152/ajpheart.1998.274.1.h233

